# Guardians and the Demand for Nurses

**Published:** 1915-03-27

**Authors:** 


### GUARDIANS AND THE DEMAND FOR NURSES.

Two statements made by Dr. Fuller, the Medical Inspector of the Local Government Board, in the course of a conference with the Scarborough Board of Guardians on the nursing staff of their workhouse infirmary, have been widely quoted and commented upon in the newspapers. Both are examples of the half-truth which is more misleading than any statement wholly erroneous. In advocating an increase of the probationer staff he suggested that, having regard to the shortage of nurses and the many hundreds that would be required during the next year or two on account of the war, it would be a patriotic act on the part of Boards of Guardians to take in hand the training of a large number of probationer nurses. The cost of the additional probationers would, he asserted, be entirely covered by discontinuing the use of beef-tea, which was of practically no nutritive value. We do not doubt that Dr. Fuller, with his ripe experience and knowledge of infirmary requirements, was right in recommending an increase in the nursing staff at Scarborough, nor can there be any dispute as to the present shortage of nurses and the increased demand for them during the course of the war. The error lies in the suggestion that by adopting the recommendation the Guardians would be helping to solve the difficulties presented by the sudden increased demand for trained nurses—a demand likely to be at its height in a few months, and not likely to last more than two or three years. No one believes that a nurse can be trained in less than two years—most teachers would say three or even four. But the demand is for the nurses now and during the next six months. Moreover, since the demand is likely to be a temporary one, care should be taken to avoid, as far as possible, meeting it by enlisting those whose livelihood would depend upon its maintenance. We believe the demand can be met in two ways: by releasing many trained nurses now employed by private individuals and public authorities on work which for the time being is less necessary, and by a system of intensive training of women who realise the temporary character of the work, and will not necessarily look to nursing as a career. Three or four months' special training, carefully planned with a view to the special circumstances of the case, in a large hospital or infirmary, would produce "nurses" who would be of real value if directed by fully trained and experienced women.

Dr. Fuller's remarks on the value of beef-tea will not receive endorsement from most medical men. Beef-tea has a very important part in the dietary of the invalid. Its actual nutritive value may be comparatively small, but if properly prepared it is far from having none. In matters of diet, moreover, nutritive value is not the only factor to be considered If it were, we should feed our sick on tabloids of proteid, fats, and carbohydrates. The stimulating properties of the extractives contained in beef-tea are of great value. Of still greater importance is the fact that it acts as a vehicle enabling a patient to take bread or other nutritious substances. Anyone who has been confined to a liquid diet knows how grateful a change a cup of beef-tea is after a course of more nutritious but less palatable beverages, how it freshens the palate and aids the taking of the diet. Even the special use Dr. Fuller made of his dictum in his argument was shown to be erroneous. The Guardians produced figures proving that the cost of beef-tea at their institution was only about a third of the estimated cost of the proposed additional probationers. The statement really had no bearing upon the desirability of increasing the staff, but its disproof no doubt influenced the Guardians in deciding to appoint one additional charge nurse instead of the six probationers advised by Dr. Fuller.

